# Tuberculosis related disability: a systematic review and meta-analysis

**DOI:** 10.1186/s12916-021-02063-9

**Published:** 2021-09-09

**Authors:** Kefyalew Addis Alene, Kinley Wangdi, Samantha Colquhoun, Kudakwashe Chani, Tauhid Islam, Kalpeshsinh Rahevar, Fukushi Morishita, Anthony Byrne, Justin Clark, Kerri Viney

**Affiliations:** 1 Faculty of Health Sciences, Curtin University, Kent St, Bentley, Perth, 6102 Western Australia Australia; 2 Telethon Kids Institute, 15 Hospital Ave, Nedlands, Perth, Western Australia 6009 Australia; 3 Research School of Population Health, The Australian National University, 62 Mills Road, Acton, Canberra, ACT 2601 Australia; 4 World Health Organization (WHO) Regional Office for the Western Pacific, The Philippines, Manila, Philippines; 5 St Vincent’s Hospital, Sydney, 406 Victoria St, Darlinghurst, Sydney, 2010 New South Wales Australia; 6 The University of New South Wales, Randwick, Sydney, 2031 New South Wales Australia; 7 Institute for Evidence-Based Healthcare, Bond University, 14 University Drive, Robina, 4266 Queensland Australia; 8 Karolinska Institutet, Solnavägen 1, 171 77 Solna, Stockholm, Sweden; 9 The University of Sydney, University Road, Camperdown, Sydney, 2066 New South Wales Australia

**Keywords:** Tuberculosis, Meta-analysis, Disability, Treatment, Impairment

## Abstract

**Background:**

The sustainable development goals aim to improve health for all by 2030. They incorporate ambitious goals regarding tuberculosis (TB), which may be a significant cause of disability, yet to be quantified. Therefore, we aimed to quantify the prevalence and types of TB-related disabilities.

**Methods:**

We performed a systematic review of TB-related disabilities. The pooled prevalence of disabilities was calculated using the inverse variance heterogeneity model. The maps of the proportions of common types of disabilities by country income level were created.

**Results:**

We included a total of 131 studies (217,475 patients) that were conducted in 49 countries. The most common type of disabilities were mental health disorders (23.1%), respiratory impairment (20.7%), musculoskeletal impairment (17.1%), hearing impairment (14.5%), visual impairment (9.8%), renal impairment (5.7%), and neurological impairment (1.6%). The prevalence of respiratory impairment (61.2%) and mental health disorders (42.0%) was highest in low-income countries while neurological impairment was highest in lower middle-income countries (25.6%). Drug-resistant TB was associated with respiratory (58.7%), neurological (37.2%), and hearing impairments (25.0%) and mental health disorders (26.0%), respectively.

**Conclusions:**

TB-related disabilities were frequently reported. More uniform reporting tools for TB-related disability and further research to better quantify and mitigate it are urgently needed.

**Prospero registration number:**

CRD42019147488

**Supplementary Information:**

The online version contains supplementary material available at 10.1186/s12916-021-02063-9.

## Background

Tuberculosis (TB) is a significant cause of death and disability worldwide, killing approximately 1.2 million people of an estimated 10 million new cases in 2019 [1]. While disability is a recognized consequence of TB, the prevalence of TB-related disability has not been estimated.

Disability includes any impairment or activity limitation as well as participation restriction [2]. Globally, low- and middle-income countries account for almost two-thirds of years lived with a disability [3]. While not well reported in the literature, TB can result in either temporary or permanent disability, arising from the disease process itself or side effects related to TB treatment, particularly related to second-line medicines used to treat drug-resistant (DR)-TB. TB service interruptions in high burden TB countries due to the ongoing COVID-19 pandemic may increase TB-related morbidity, disability, and mortality [4, 5].

Physical disabilities related to TB vary according to the bodily site affected by TB. For example, people with a history of pulmonary TB may suffer from a range of long-lasting respiratory-related sequelae such as impaired lung function (obstructive, restrictive, reduced diffusing capacity, or reduced lung volumes), chronic obstructive pulmonary disease (COPD), bronchiectasis, aspergillosis, pulmonary hypertension, or pulmonary fibrosis [6–9]. The global burden of COPD as a consequence of TB has recently been estimated to be 5.9 million disability-adjusted life years (DALYs) [10]. TB of the nervous system, affecting the meninges, brain, spinal cord, or cranial and peripheral nerves, can cause severe irreversible disability [11]. For example, spinal TB can result in paraparesis and quadriparesis due to spinal deformity and damage of the neural structures, often leading to permanent physical disabilities [12]. Some disabilities arise due to organ or tissue destruction in the host from TB disease, while others are a result of adverse effects of treatment. TB treatment is effective, prevents death, and limits disability, but certain medications have side effects which may result in temporary or permanent disability. Previous studies have demonstrated an increased prevalence of visual disturbance and hearing loss among people previously treated for DR-TB [13, 14]. However, some of the medicines that were used in these studies such as kanamycin and capreomycin are no longer recommended by the World Health Organization [15].

Mental health disorders may also be more prevalent among TB survivors than the general population [16, 17]. Mood disorders including anxiety and depression may be associated with TB disease, TB treatment, or factors not directly related to TB. Whereas the long treatment duration of 9–20 months for DR-TB results in disruptions to usual work, family, and social activities, TB patients may also be subjected to stigma and discrimination due to cultural norms or beliefs associated with TB, which can cause or exacerbate mental health disorders. Although not well studied, the effect of TB treatment on the cognitive development of children and adolescents as a result of disruption to schooling may also be significant [18].

Despite a growing interest in the long-term sequelae associated with TB, the global prevalence of TB-related disability is currently unknown. Describing the spectrum and prevalence of TB-related disabilities is crucial to inform service provision and policy making in countries where TB is common and to mitigate future disability in patients being treated for TB. In this systematic review, we aimed to quantify the global prevalence and types of TB-related disabilities.

## Methods

### Search strategy and selection criteria

We performed a systematic review following the Preferred Reporting Items for Systematic Reviews and Meta-Analyses (PRISMA) guidelines [19]. We searched PubMed, Embase, and Web of Science databases for studies that reported on permanent disability associated with TB, using pre-specified search terms. We checked the reference lists of included papers for additional relevant references and performed a backwards and forwards citation search. Our search strategy (Additional file 1) was developed by a senior research information specialist (JC) and respiratory physician (AB), both with extensive experience in conducting systematic reviews in health and medicine.

The screening of articles by title and abstract was carried out independently by three researchers (KW, KAA and SC) in Rayyan [20]. Full-text papers were then independently screened by four researchers (KW, KAA, CK, and SC) using eligibility criteria described below. Disagreement was resolved through discussion and consensus.

#### Inclusion criteria

Participants were people in all age groups with any type of TB (pulmonary and extra pulmonary TB, new and relapse, drug-sensitive (DS), and DR-TB), from all regions and countries. Our intervention of interest was TB treatment based on national and international guidelines for TB (for both DS and DR-TB). However, studies without a specific intervention (i.e., for those who did not specifically report that the patients were on TB treatment but which clearly stated that the patients had been diagnosed with TB) were also included.

Our outcomes of interest were the prevalence of TB patients who developed a permanent form of disability, detected or reported after TB diagnosis, and where TB disease or TB treatment may have contributed to the disability. Our definitions of disability are included in Additional file 2.

We included observational studies (e.g., cross-sectional, case-control, or cohort studies) and experimental epidemiological studies that reported data from the year 2000 until July 2019. Our research question in the PICO (Population, Intervention, Comparator, Outcome) format is included in Table 1.

**Table 1:** Research question formulated in the Population, Intervention, Comparator, and Outcome format for a systematic review on disabilities associated with tuberculosis

Population/Participants	Intervention	Comparator	Outcome
Patients with TB: -DS and DR-TB -Adults and children -Pulmonary and extra-pulmonary -All countries (i.e., global focus, low, middle- and high-income countries with low and high incidence of TB)	Receiving treatment for TB (DS or DR-TB)	No comparator	Physical or mental health disability (irreversible or long term), related to the disease process and/or TB treatment

*DS* drug susceptible, *DR* drug resistant, *TB* tuberculosis

#### Exclusion criteria

We excluded studies that reported temporary disabilities (for example, a mental health disorder attributable to an adverse event during treatment that was resolved by a change of medication). Descriptive epidemiological studies were also excluded (case reports and case series) as were other systematic reviews; scientific correspondence, posters, and conference abstracts; studies conducted in animals; and historical data reported before the year 2000.

### Data extraction and quality assessment

Data were extracted into a Microsoft Excel 2016 spreadsheet (Microsoft, Redmond, Washington, USA) by four researchers (KW, SC, CK, and KA). The data extraction spreadsheet was pilot tested and refined before extraction. The lists of variables included in the data extraction tool are available in the Additional file 3. All included articles were assessed for quality using a modified Ottawa Newcastle quality assessment scoring tool [21].

### Data analysis

Meta-analysis was performed to estimate the pooled prevalence of each form of disability using the inverse variance heterogeneity model. Stratified analyses were conducted by country income-level and TB type, separately for each disability when two or more studies were available on the outcome of interest (see Additional file 4 for details).

This review was registered in the Prospective Register of Systematic Reviews (PROSPERO, CRD42019147488). Ethical approval was not sought for this study as it includes an analysis of secondary data.

## Results

### Characteristics of the included studies

**Fig. 1:**
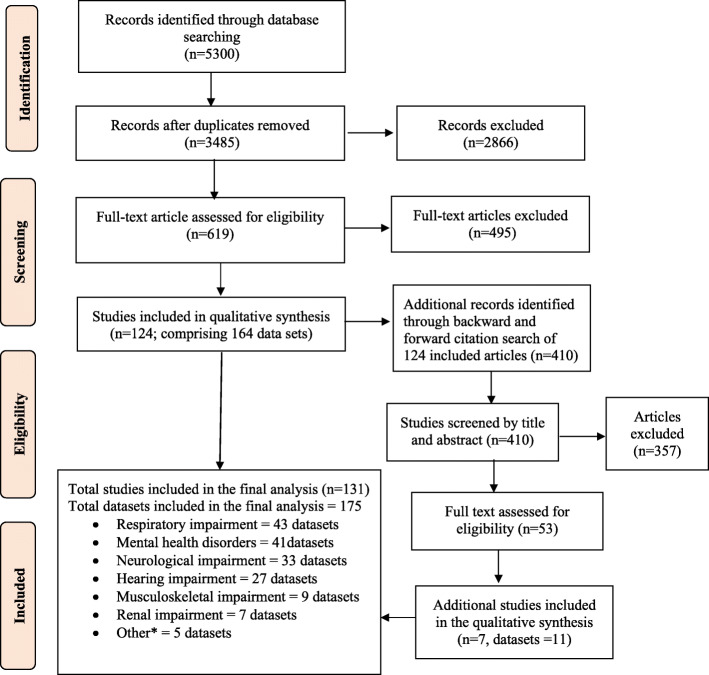
Study identification and selection flow chart. *Others include hypothyroidism, diabetes, carcinoma, endocrinopathies, and hepatic failure

The search strategy yielded 3485 unique publications, 619 articles remained after the title and abstract screening. After full-text review, 124 publications (comprising 164 datasets) were included in the review. A backward and forward citation search found 410 publications, of which 53 were not identified in the original search; seven of these were subsequently included in the final analysis. Some studies reported more than one type of disability, and thus a total of 175 datasets (217,475 patients) from 131 unique studies were included (Fig. 1).

The characteristics of the included studies are presented in Table 2. The included studies were conducted in 49 countries. The majority of studies were conducted in India 24.6% (*n*=43) followed by South Africa 9.7% (*n*=17) and Brazil 5.1% (*n*=9). The mean age of study participants was 36.7 years (±16.3) and 59.6% of cases were male. More than one-third of studies (39%, *n*=68) reported that their study population had DS-TB, while 29.1% (*n*=51) included patients with DR-TB. The site of TB disease was not reported in 41.1% of studies (*n*=72), while 28.6% (*n*=50) and 16.0% (*n*=28) included patients with pulmonary TB (PTB) and extra-pulmonary TB (EPTB), respectively. More than half of the studies (52.9%, *n*=90) reported that disabilities were diagnosed during TB treatment.

**Table 2:** Characteristics of included studies

First author	Publication year	Country	Country income level	Type of TB	Years of data collection	Study design	Male proportion	Mean age	Sample size
*Hearing impairment*									
Seddon [22]	2012	South Africa	UMI	DR	2009–2010	Retrospective cohort	48.0	3.6	94
Shean* [23]	2013	South Africa	UMI	DR	2002–2008	Retrospective cohort	53.9		115
Ghafari [24]	2015	South Africa	UMI	DR	2010	Prospective cohort	45.0	7	25
Sagwa [25]	2015	Namibia	UMI	DR	2004–2014	Retrospective cohort	56.09	36.4	353
Appana [26]	2016	South Africa	UMI	DR	2016	Prospective cohort	52.0	34	52
Khoza-Shangase [27]	2016	South Africa	UMI	DS & DR (I)	2012–2014	Retrospective cohort	46.0	36.6	191
Trebucq* [28]	2018	Multiple countries^†^	LI	DR	2013–2015	Prospective cohort	66.3	34	1006
Harouna [29]	2019	Niger	LI	DR	2008–2013	Retrospective	70.0	17	10
Cohen [30]	2019	Malawi	LI	DS & DR (I)	2013–2014	Prospective cohort	64.6	37	158
Shibeshi [31]	2019	Ethiopia	LI	DR	2010–2015	Retrospective cohort	54.2	32	879
Bloss [32]	2010	Latvia	UMI	DR	2000–2003	Retrospective cohort	76.0	42	996
Ribeiro [33]	2015	Portugal	HI	DR	2009–2012	Prospective	36.4	41	10
Batirel [34]	2015	Multiple countries^‡^	UMI	DS	2000–2013	Retrospective cohort	52.0	51	314
Lima [35]	2006	Brazil	UMI	DS & DR (I)	2000–2001	Cross-sectional	79.4	38.8	36
Vasconselos [36]	2017	Brazil	UMI	DR	2006–2014	Retrospective	53.0		172
Kittikraisak [37]	2008	Thailand	LMI	DS & DR (I)	2005–2008	Prospective	70.0	35	493
Bharat [38]	2014	India	LMI	DR	2012–2013	Retrospective cohort	63.28	42	207
Nataprawira* [39]	2016	Indonesia	LMI	DS	2007–2010	Prospective cohort	55.2	3.7	29
Prasad* [40]	2016	India	LMI	DR	2009–2010	Prospective cohort	69.4	29.3	98
Sharma [41]	2016	India	LMI	DR	2012	Prospective	68.0	37.5	100
Synmon* [42]	2017	India	LMI	DS & DR (NI)	2013–2015	Prospective cohort	61.3	32.3	93
Justin [43]	2019	India	LMI	DR	2006–2015	Retrospective cohort	46.7	29	30
Piparva [44]	2018	India	LMI	DR	2014–2015	Retrospective cohort	66.7	32.8	108
Hoa [45]	2015	Vietnam	LMI	DR	2010	Cross-sectional	65.0		282
Lebogang [46]	2012	South Africa	UMI	DR	~ 2011	Cross-sectional	49.0	33	53
Singla* [47]	2009	India	LMI	DR	2002–2006	Prospective cohort	53.9		126
Aznar* [48]	2019	Angola	UMI	DR	2013–2015	Prospective cohort	57.4		216
*Mental health disorders*									
Issa [49]	2009	Nigeria	LI	-	2008	Retrospective cohort	63.1	35.1	65
Deribew [50]	2010	Ethiopia	LI	DS	2009	Case control	41.8	33.4	620
Ige [51]	2011	Nigeria	LI	DS	2010	Prospective cohort	31.8	27.1	88
Shean* [23]	2013	South Africa	UMI	DR	2002–2008	Retrospective cohort	53.9		115
van den Heuvel [52]	2013	Zambia	LMI	DS	2009–2010	Cross-sectional	54.0	33.9	231
Peltzer [53]	2013	South Africa	UMI	DS	2011	Cross-sectional	54.5	36.2	4225
Peltzer [54]	2013	South Africa	UMI	DS	2011	Cross-sectional	54.5	36.1	4900
Xavier [55]	2015	Angola	UMI	DS & DR (I)	2013–2015	Cross-sectional	58.0		18
Duko [56]	2015	Ethiopia	LI	DS	2014	Prospective		34.5	417
Kehbila [57]	2016	Cameroon	LMI	DS	2015	Cross-sectional	49.7	36.9	265
Ambaw [58]	2017	Ethiopia	LI	DS	2014–2016	Cross-sectional	54.2	30	657
Tomita [59]	2019	South Africa	UMI	DR	2015–2016	Prospective cohort	22.0		141
Dasa [60]	2019	Ethiopia	LI	DS & DR (I)	2017	Cross-sectional	59.0	39	403
Aamir [61]	2010	Pakistan	LMI	DS & DR (I)	2007–2008	Prospective			65
Hadadi* [62]	2010	Iran	UMI	DS	2003–2005	Retrospective	61.3	39.8	403
Kaukab [63]	2015	Pakistan	LMI	DR	2014	Randomized control trial	45.7		70
Tariq [64]	2018	Pakistan	LMI	DS	2017	Case control	59.6		151
Khan [65]	2018	Pakistan	LMI	DR	2016–2017	Cross-sectional	52.0	31	1279
Bloss [32]	2010	Latvia	UMI	DR	2000–2003	Retrospective cohort	76.0	42	996
Yilmaz [66]	2016	Turkey	UMI	DS	2014–2015	Cross-sectional	63.0	45.5	208
Soriano-Arandes* [67]	2019	Spain	HI	DS & DR (I)	2005–2013	Retrospective cohort	50.7	1.1	134
dos-Santos [68]	2017	Brazil	UMI	-	2013	Cross-sectional	69.8	44.6	86
Castro-Silva [69]	2018	Brazil	UMI	DS	2015–2016	Cross-sectional	62.6	40.7	98
Bharat [38]	2014	India	LMI	DR	2012–2013	Retrospective cohort	63.28	42	207
Pardal [70]	2015	India	LMI	DS	2014–2015	Case control	100.0		100
Galhenage [71]	2016	Sri Lanka	LMI	DS	2014–2015	Cross-sectional	73.0	46.4	430
Prasad* [40]	2016	India	LMI	DR	2009–2010	Prospective cohort	69.4	29.3	98
Akaputra [72]	2017	Indonesia	LMI	DS	2016	Cross-sectional	74.5		55
Salodia [73]	2019	India	LMI	DS & DR (I)	2018	Cross-sectional	57.5	38.4	106
Masumoto [74]	2014	Philippines	LMI	DS	2012	Cross-sectional	65.4	41.9	561
Shen [75]	2014	Taiwan	HI	-	2000–2001	Case control	67.8	60.9	9092
Lee [76]	2017	Taiwan	HI	-	2013–2014	Cross-sectional	65.5	65.2	84
Xu [77]	2017	China	UMI	DS		Cross-sectional	70.5	53.6	372
Gong [78]	2018	China	UMI	-	2013–2014	Cross-sectional	67.4	47.7	1342
Singla* [47]	2009	India	LMI	DR	2002–2006	Prospective cohort	53.9	26	126
Aznar* [48]	2019	Angola	UMI	DR	2013–2015	Prospective cohort	57.4	30	216
*Musculoskeletal impairment*									
Hadadi* [62]	2010	Iran	LMI	DS	2003–2005	Retrospective	61.3	39.8	403
Tinsa [79]	2019	Tunisia	LMI	DS	2005–2007	Retrospective cohort	41.5	7.5	41
Sezgi [80]	2014	Turkey	UMI	DS	2005–2010	Retrospective cohort	60.9		21
Batirel [34]	2015	Multiple countries^‡^	UMI	DS	2000–2013	Retrospective cohort	52.0	51	314
Soriano-Arandes* [67]	2019	Spain	HI	DS & DR (I)	2005–2013	Retrospective cohort	50.7	1.1	134
Samuel [81]	2011	India	LMI	DS	2003–2008	Retrospective cohort	68.7	38	16
Kamara* [82]	2013	India	LMI	DS	2011	Cross-sectional	47.0	34	228
Agarwal [83]	2017	India	LMI	DS	2010–2015	Retrospective	40.0	8.2	30
Luo [84]	2018	China	UMI	DS	2009–2015	Retrospective	57.7	38.38	189
*Neurological impairment*									
Njoku [85]	2007	Nigeria	LI	DS	2000–2004	Prospective	77.2		92
Trebucq* [28]	2018	Multiple countries^†^	LI	DR	2013–2015	Prospective cohort	66.3	34	1006
Cohen [30]	2019	Malawi	LI	DS & DR (I)	2013–2014	Prospective cohort	64.6	37	158
Benzagmout [86]	2011	Morocco	LMI	DS	2001–2006	Retrospective cohort	64.9	9.1	37
Shaikh [87]	2012	Pakistan	LMI	DS	2006–2011	Retrospective cohort	52.0	37.7	50
Barungi [88]	2014	South Africa	UMI	DS	2009	Retrospective	50.0	2.7	36
Quereshi [89]	2013	Pakistan	LMI	DS & DR (I)	2001–2010	Retrospective	57.5	36	87
Alper [90]	2008	Turkey	UMI	DS & DR (I)	2000–2004	Retrospective cohort	58.3	34.5	12
Bloss [32]	2010	Latvia	UMI	DR	2000–2003	Retrospective cohort	76.0	42	996
Christensen [91]	2011	Denmark	HI	DS & DR (I)	2000–2008	Retrospective cohort	48.0	30	50
Miftode [92]	2015	Romania	UMI	DS & DR	2004–2013	Retrospective cohort	59.0	29.3	204
Batirel [34]	2015	Multiple countries^‡^	UMI	DS	2000–2013	Retrospective cohort	52.0	51	314
Paulsrud [93]	2019	Denmark	HI	DS & DR (I)	2000–2015	Retrospective	29.0	4	21
Soriano-Arandes* [67]	2019	Spain	HI	DS & DR (I)	2005–2013	Retrospective cohort	50.7	1.08	134
Lucena [94]	2015	Brazil	UMI	DS	2010–2013	Cross-sectional	79.2	50.8	24
Ramos [95]	2017	USA	HI	DS	2003–2011	Retrospective cohort	61.0	51	2789
Karande [96]	2005	India	LMI	DS	2000–2003	Prospective		3.1	123
Kalita [97]	2007	India	LMI	DS	2003–2006^¥^	Prospective cohort	58.5	33.2	65
Wani [98]	2008	India	LMI	DS	2004–2007^¥^	Prospective	40.0		38
Garg [99]	2010	India	LMI	DS	2005–2007	Prospective cohort	53.0	26	60
Gunawardhana [100]	2012	Sri Lanka	LMI	DS	2010–2011	Prospective cohort	63.0	44	89
Lisha* [101]	2012	India	LMI	DS	2008–2010	Cross-sectional	81.0	47	224
Kamara* [82]	2013	India	LMI	DS	2011	Cross-sectional	47.0	34	228
Nataprawira* [39]	2016	Indonesia	LMI	DS	2007–2010	Prospective cohort	55.2	3.7	29
Synmon [42]	2017	India	LMI	DS & DR (NI)	2013–2015	Prospective cohort	61.3	32.3	93
Justin [43]	2019	India	LMI	DR	2006–2015	Retrospective cohort	46.7	29	30
Sheu [102]	2010	Taiwan	HI	DS	2000–2003	Retrospective cohort	63.9		2283
Chen [103]	2014	Taiwan	HI	DS	2002–2006	Prospective	61.5	65.1	38
Chen [104]	2015	Taiwan	HI	-	2009–2010	Case control	76.5	50.8	17
Hoa [45]	2015	Vietnam	LMI	DR	2010	Cross-sectional	65.0		282
Shen [105]	2016	Taiwan	HI	-	2000–2009	Retrospective cohort	71.9	63	100000
Luo [84]	2018	China	UMI	DS	2009–2015	Retrospective cohort	57.7	38.38	189
Aznar* [48]	2019	Angola	UMI	DR	2013–2015	Prospective	57.4	30	216
Sheu [102]	2010	Taiwan	HI	-	2000–2003	Retrospective cohort	-	-	2283
*Renal impairment*									
Shean* [23]	2013	South Africa [106]	UMI	DR	2002–2008	Retrospective cohort	53.9		115
Arnold [107]	2017	UK	HI	DR	2008–2014	Prospective			8
Ramos [95]	2017	USA	HI	-	2003–2011	Retrospective cohort	61.0	51	2789
Wagaskar [108]	2016	India	LMI	DS	2011–2013		58.1	36.2	31
Aznar* [48]	2019	Angola	UMI	DR	2013–2015	Prospective	57.4	30	216
*Respiratory impairment*									
Issa [49]	2009/10	Nigeria	LMI	-	2008	Prospective cohort	63.1	35.1	67
Maydell [109]	2010	South Africa	UMI	DS	2004–2007	Retrospective cohort	38.1	1.7	21
Ngahane [110]	2015	Cameroon	LMI	DS	2014	Cross-sectional	54.3	34.2	269
Manji [106]	2016	Tanzania	LI	DS	2014	Cross-sectional	60.5		501
Chin [111]	2018	Zimbabwe	LMI	DS & DR (I)	2011–2016	Prospective cohort		41	175
Fiogbe [112]	2019	Benin	LI	DS	2016	Cross-sectional	67.7	37	189
Mkoko [113]	2019	South Africa	UMI	DS	2016	Retrospective	50.8	50.8	173
Cohen [30]	2021	Malawi	LI	DS & DR (I)	2013–2014	Prospective cohort	64.6	37	158
Baig [114]	2010	Pakistan	LMI	-	2007	Prospective cohort	76.5	53.4	47
Radovic [115]	2016	Serbia	UMI	DS	2005–2012	Case control	80.0	58.8	40
Soriano-Arandes* [67]	2019	Spain	HI	DS & DR (I)	2005–2013	Retrospective cohort	50.7	1.1	134
Vashakidze [116]	2019	Georgia (Tbilisi)	LMI	DR	2009–2011	Cross-sectional	57.0	31.2	58
Ramos [117]	2006	Brazil	UMI	DS	2000–2004	Retrospective cohort		30	218
Morrone [118]	2007	Brazil	UMI	DS	2003	Prospective	66.6	35.2	75
Byrne [119]	2017	Peru	UMI	DS & DR (I)	2014	Prospective cohort	57.6	29	177
Godoy [120]	2012	Brazil	UMI	DR	2008–2010	Cross-sectional	67.0	43.7	18
Nihues [121]	2015	Brazil	UMI	-	2002–2012	Cross-sectional	52.1	40	121
Maguire [122]	2009	Indonesia	LMI	DS	2003–2004	Prospective cohort	66.7	29.1	69
Singla [47]	2009	India	LMI	DR	2009	Cross-sectional	55.6	33.5	51
Bhattacharyya [123]	2011	India	LMI	-	2006–2010	Retrospective cohort			161
Lisha* [101]	2012	India	LMI	DS	2008–2010	Cross sectional	81.0	47	224
Das [124]	2014	India	LMI	DR	2012–2014	Retrospective cohort	57.1	34.7	45
Gandhi [125]	2016	India	LMI	DS	2013	Case control	71.8		146
Panda [126]	2016	India	LMI	-		Cross-sectional	71.3	38	101
Deepak [127]	2017	India	LMI	-	2016	Case control	88.9	60.2	74
Mukati [128]	2017	India	LMI	DR	2014	Prospective cohort	70.0	36.8	130
Santra [129]	2017	India	LMI	DS	2014–2015	Cross sectional	84.1	53.4	218
Patil [130]	2018	India	LMI	DS	2013–2017	Prospective	60.1		1000
Singla [131]	2018	India	LMI	DR	2002–2006	Prospective	54.3	27.6	46
Gupte [132]	2019	India	LMI	DS	2016–2019	Prospective cohort	52.0	32	172
Lee [133]	2003	Republic of Korea	HI	DS		Prospective	56.0	65.2	11
Lam [134]	2010	China	UMI	DS	2003–2006	Retrospective cohort	26.4	61.9	1954
Hwang [135]	2014	Republic of Korea	HI	DS	2001–2002	Prospective	45.4	51	1384
Rhee [136]	2013	Republic of Korea	HI	DS	2005–2012	Retrospective cohort	60.5	65.6	457
Jung [137]	2015	Republic of Korea	HI	-	2008–2012	Prospective cohort	43.3	57.1	14967
Jo [138]	2017	Republic of Korea	HI	DS	2010–2015	Retrospective			195
Jianmin [139]	2018	China	UMI	DS	2008–2016	Retrospective cohort	67.5	76.8	104
Park [140]	2018	Republic of Korea	HI	DS	2011–2017	Retrospective cohort	85.6	73.2	182
Sun [141]	2018	China	UMI	DS	2013–2016	Retrospective cohort	49.6	34.5	135
Akkara [142]	2013	India	LMI	DS	2011–2012	Prospective cohort	74	-	257
*Visual impairment*									
Shean* [23]	2013	South Africa	UMI	DR	2002–2008	Retrospective cohort	53.9		115
Bloss [32]	2010	Latvia	UMI	DR	2000–2003	Retrospective cohort	76.0	42	996
Urzua [143]	2017	Chile; Spain	HI	DS	2002–2012	Retrospective cohort	25.7	54.9	35
Gunasekeran [144]	2018	UK	HI	DS	2007–2014	Retrospective cohort	53.4	48.5	354
Bharat [38]	2014	India	LMI	DR	2012–2014	Retrospective cohort	63.3	42	207
Soumyava [145]	2014	India	LMI	DS	2011–2012	Retrospective cohort	67.5	34.4	61
Nataprawira* [39]	2016	Indonesia	LMI	DS	2007–2010	Prospective cohort	55.2	3.67	29
Synmon* [42]	2017	India	LMI	DS & DR (NI)	2013–2015	Prospective cohort	61.3	32.3	93
Hsia [146]	2015	Taiwan	HI	-	2000–2010	Retrospective cohort	67.9	56	6994
Others^									
Satti [147]	2011	Lesotho	LMI	DR	2007–2009	Retrospective cohort	60	-	186
Jo [138]	2017	Republic of Korea	HI	DS	2010–2015	Retrospective cohort	67	63.5	195
Lisha [101]	2012	India	LMI	DS		Cross-sectional			224
Wani [98]	2008	India	LMI	DS		Prospective cohort			38
Harouna [29]	2019	Niger	LI	DR	2008–2013	Retrospective cohort	84	31	110
Prakash [148]	2017	India	LMI		2008–2013	Prospective cohort	55	11.3	44

*AFR* African Region, *SEAR* South-East Asia Region, *EUR* European Region, *EMR* Eastern Mediterranean Region, *PAHO* Pan American Health Organization, *WPR* Western Pacific Region, *LI* low-income, *LMI* lower middle-income, *UMI* upper middle-income, *HI* high-income, *DS* drug sensitive TB, *DR* drug-resistant TB, *DS & DR (I)* drug-sensitive and drug-resistant TB with injectables for treatment, *DS & DR (NI)* drug-sensitive and drug-resistant TB with no injectable

*Indicates studies with more than one disability

^†^Burkina Faso, Burundi, Benin, Democratic Republic of Congo, Cote d’Ivoire, Cameroon, Niger, Rwanda

^‡^Turkey, Egypt, Albania, Greece

^¥^Exact year of study not given

^Others include hypothyroidism, diabetes, carcinoma, endocrinopathies, and hepatic failure

### Prevalence of disabilities

The review showed that the most common type of disabilities were mental health disorders (23.1%), respiratory impairment (20.7%), musculoskeletal impairment (17.1%), hearing impairment (14.5%), visual impairment (9.8%), renal impairment (5.7%), and neurological impairment (1.6%).

### Sources of heterogeneity

There was large heterogeneity in the prevalence of disability. Two variables, namely country income level and type of TB, were identified as the source of heterogeneity across all types of disability and therefore were used as the primary variables of stratification.

### Prevalence of disabilities by country income level

**Fig. 2:**
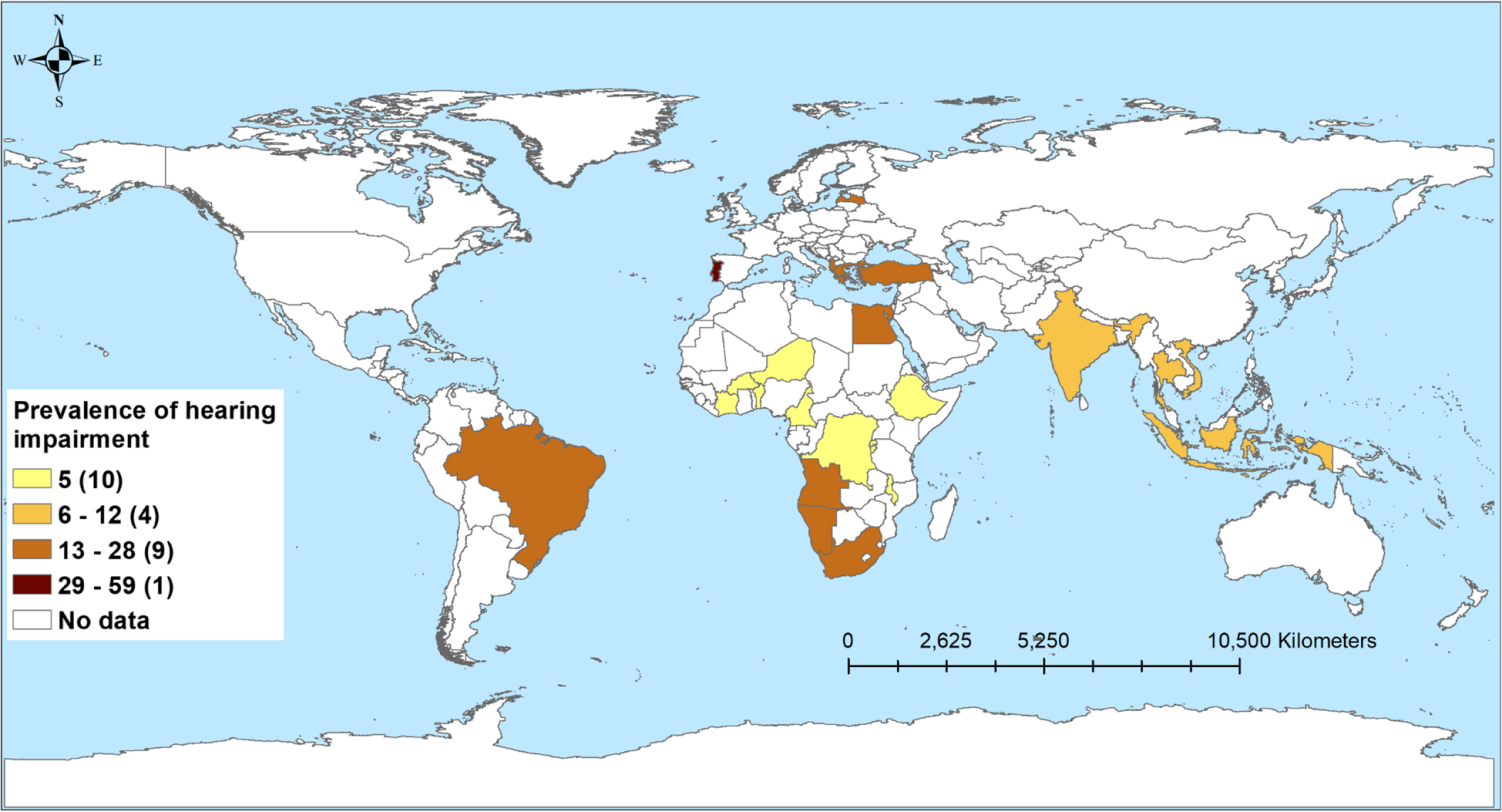
Prevalence of tuberculosis patients with hearing-related disorders from 24 studies in 23 countries

**Fig. 3:**
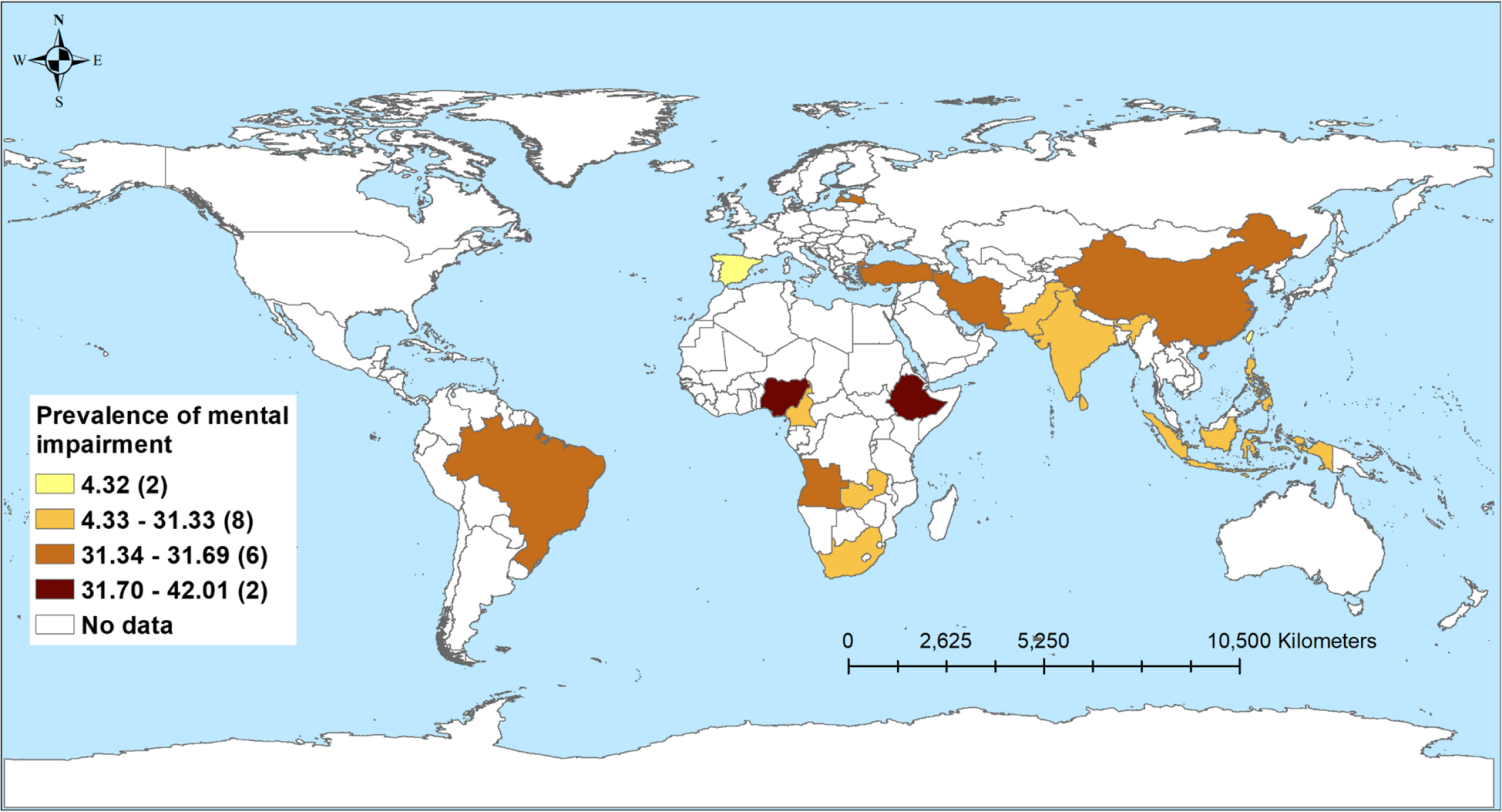
Prevalence of tuberculosis patients with mental health disorders from 39 studies in 18 countries

**Fig. 4:**
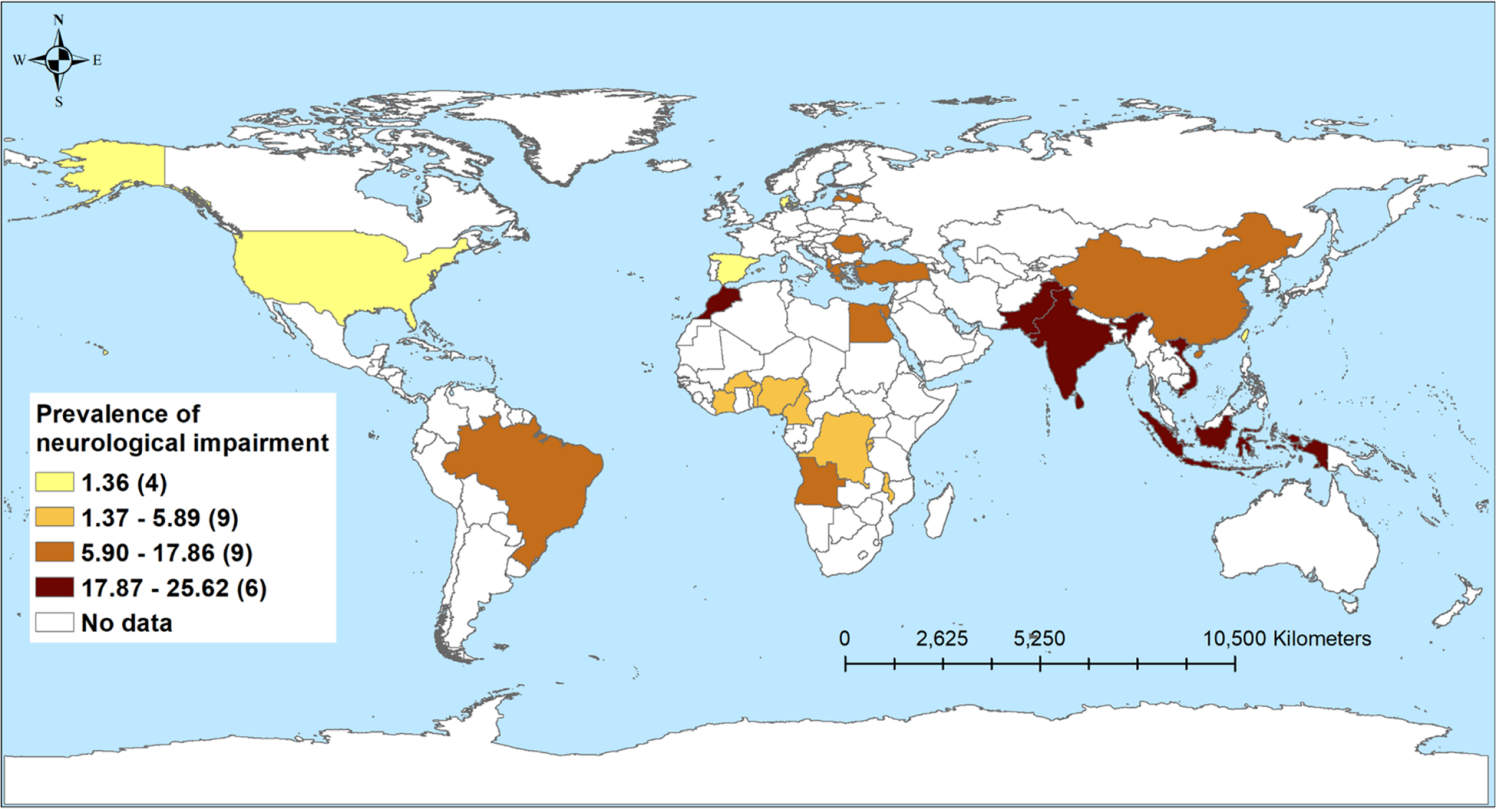
Prevalence of tuberculosis patients with neurological impairment from 31 studies in 27 countries

**Fig. 5:**
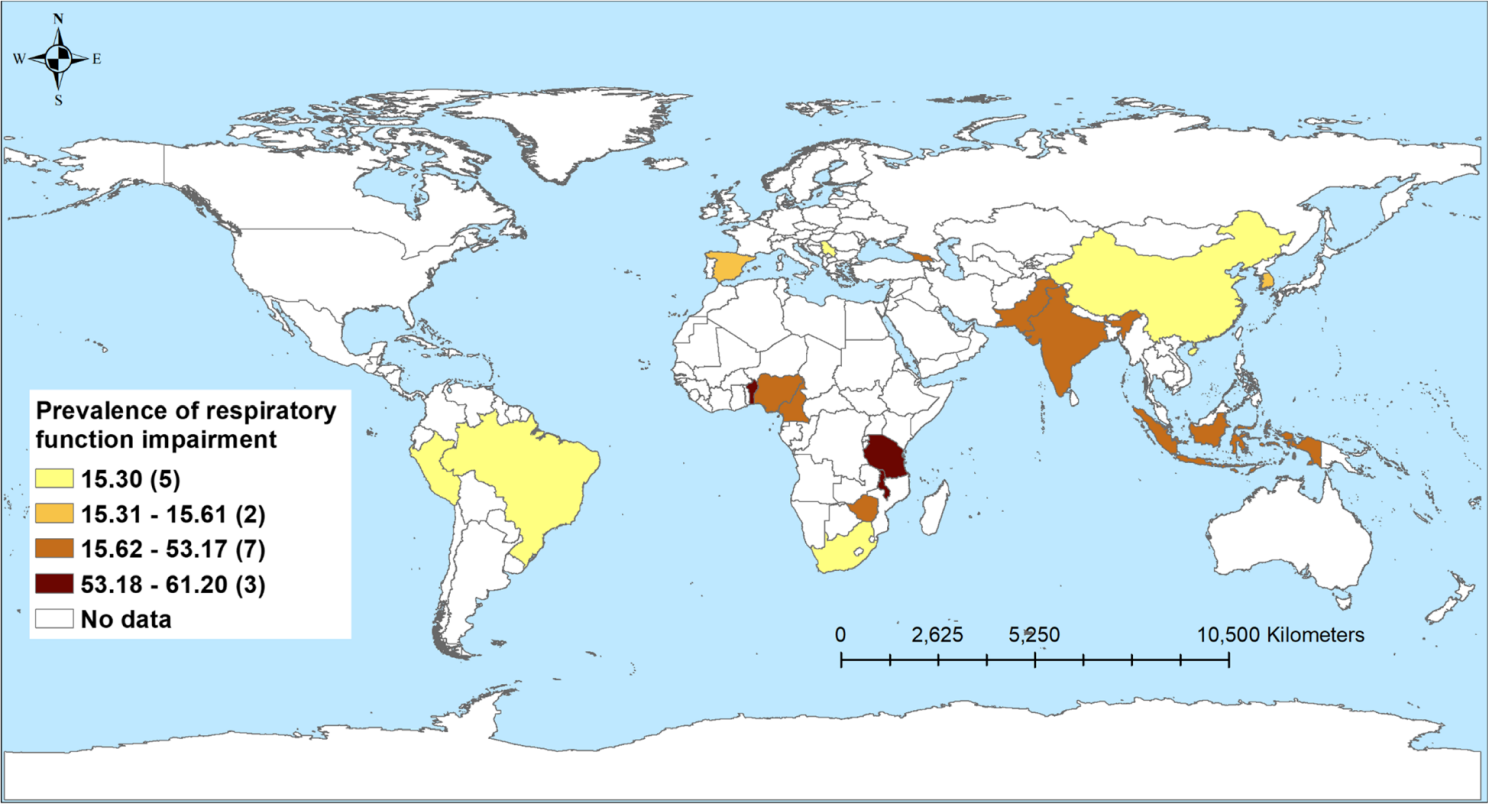
Prevalence of tuberculosis patients with respiratory impairment from 42 studies in 17 countries

Table 3 shows the number of studies and the prevalence of disabilities associated with TB, stratified by country income level. A total of 43 studies reported respiratory impairment. Nearly two-thirds of patients in LICs (61.2%) and just over half in LMICs (56.1%) experienced some form of respiratory impairment. The prevalence was low among HICs (14.9%) and UMICs (15.3%) (Fig. 2). Similarly, the highest prevalence of patients with mental health disorders was observed among patients in LICs (42%) followed by LMICs (31.3%), UMICs (30.6%), and HICs (4.3%; Fig. 3). The prevalence of patients with neurological function impairment was highest in LMICs (25.6%) and UMICs (15.9%) and lowest in LICs (5.9%) and HICs (1.3%; Fig. 4). The highest prevalence of TB patients who experienced hearing impairment (59.1%) was reported in HICs and UMICs 27.4% and 11.0% and 5% were reported from LMICs and LICs, respectively (Fig. 5).

**Table 3:** Pooled prevalence of mental health disorders, respiratory impairment, musculoskeletal impairment, hearing impairment, visual impairment, and neurological impairment, stratified by study characteristics

Categories	Respiratory impairment			Mental health disorders			Hearing impairment			Neurological impairment			Visual impairment			Musculoskeletal impairment	
	Number of studies	Prevalence of disabilities		Number of studies	Prevalence (%)		Number of studies	Prevalence of disabilities		Number of studies	Prevalence of disabilities		Number of studies	Prevalence of disabilities		Number of studies	Prevalence of disabilities
Overall	43	20.7		41	23.1		27	14.5		33	1.6		9	9.8		9	17.1
**Countries income level**																	
High-income	9	14.9		3	4.3		1	59.1		9	1.3		3	11.1		1	2.5
Upper middle-income	12	15.3		14	30.6		11	27.4		8	15.9		2	3.1		4	32.5
Lower middle-income	19	56.1		18	31.3		11	11.0		13	25.6		4	10.7		4	4.7
Low-income	3	61.2		6	42.0		4	5.0		3	5.9		-	-		-	-
**Type of TB**																	
Drug susceptible	24	33.1		24	21.9		2	2.3		17	12.5		4	11.9		8	20.2
Drug resistant	7	58.7		7	26.0		20	15.0		6	4.6		3	2.7		-	-
Drug susceptible and drug resistant (no injectable)	-	-		-	-		1	2.6		3	28.5		1	6.5		-	-
Drug susceptible and drug resistant (injectable)	4	33.1		6	20.7		3	25.0		5	37.2		-	-		1	2.5
**Study design**																	
Cohort	28	18.1		15	23.2		24	14.5		28	1.5		9	9.8		8	23.0
Cross-sectional	10	54.5		22	33.3		3	13.6		4	6.8		-	-		1	2.7
Case control	5	47.9		4	05.0		-	-		1	30.5		-	-		-	-
**HIV** (%)																	
< 1	5	14.7		5	4.9		3	2.7		5	9.0		1	1.1		3	21.2
1–50	9	51.8		12	31.5		10	12.5		11	8.2		3	3.3		1	38.2
51–100	3	38.9		6	30.0		3	22.5		12	65.1		.	-		-	-
Not recorded	26	18.8		18	38.5		11	26.4		16	1.3		5	11.5		5	12.8
**Timing of disability diagnosis**																	
Before TB treatment	9	17.6		1	64.2		-	-		6	8.9		1	75.0		1	36.9
During TB treatment	6	61.0		35	22.0		22	14.7		11	1.23		4	3.0		6	12.4
After TB treatment	27	19.3		2	19.8		4	14.1		14	40.9		4	11.1		2	4.0
Not recorded	1	63.2		3	44.7		1	4.3		2	5.7		-	-		-	-

Dash line (-) indicates that there was no available study for the sub-group analysis

### Prevalence of disabilities by the type of TB

Among patients with DS-TB, the prevalence of respiratory impairment was 33.1% while 21.9% of patients reported mental disorders, 12.5% reported neurological impairment, 11.9% reported visual impairment, and 2.3% reported hearing impairment (Table 3). Among patients with DR-TB, the prevalence of patients reporting respiratory impairment was 58.7% while 26% reported mental disorder, 15% reported hearing impairment, 4.6% reported neurological impairment, and 2.7% reported visual impairment. Studies including patients with DS-TB and DR-TB (with the inclusion of an injectable agent) reported that 37.2% of patients had a neurological impairment, 33.1% had respiratory impairment, 25% had a hearing impairment, and 20.7% had a mental disorder, with none of the studies reporting visual impairment. Neurological impairment was also high in studies which included DS-TB and DR-TB patients who did not receive an injectable agent (28.5%). Additional information on disabilities by HIV status, timing of disability diagnosis, and study design is provided in Table 3.

### Quality assessment

The quality of included studies was low to moderate overall, with a median score of 5 points (the maximum score is 9 points). Of the included 131 studies, only 11 studies had a score of 8 or 9 points, regarded as high-quality studies. The remaining studies scored 7 points or less, with 28 of them scoring 4 points or lower, classified as a low-quality study. Additional file 5: Table S1 presents the results of the quality assessment scores and Additional file 6: Table S2 includes the quality assessment tools.

## Discussion

This systematic review attempts to quantify the prevalence and types of TB-related disabilities. We found a substantial burden of TB-related disabilities, with four common types: (1) respiratory impairment, (2) hearing impairment, (3) mental health disorders, and (4) neurological impairment.

### Respiratory impairment

Respiratory impairment was the most common disability identified in this review. There was inconsistency in how respiratory impairment was diagnosed and reported. However, the prevalence of respiratory impairment was heterogeneous when stratified by country income level. The highest prevalence was reported in LICs (61.2%) and LMICs (56.1%). This may correlate with the high burden of TB in these countries, difficulties in accessing health care, or poverty. Poverty is widely recognized as a risk factor for TB and may also result in respiratory impairment [149–151]. High rates of respiratory impairment in LICs and LMICs may also be partially explained by low levels of health service coverage [152, 153]. High coverage of essential health services, including early access to TB diagnosis, treatment, and care, with appropriate monitoring of patients while on treatment, may minimize the long-term sequelae and disabilities associated with TB [154]. Other causes of lung diseases such as cigarette smoking and air pollution (indoor and outdoor) may contribute to the high prevalence of respiratory disabilities observed in LICs and LMICs [155, 156]. One systematic review reported a positive association between a history of TB treatment and chronic respiratory diseases, including COPD and bronchiectasis [157]. This association was much stronger in non-smokers and in high TB incidence countries [157]. WHO has recommended an integrated strategy to manage respiratory patients in primary health care settings with a focus on priority respiratory diseases, particularly TB [158].

Respiratory impairment was also higher among those with DR-TB. We found an almost twofold increase in the prevalence of respiratory impairment among patients with DR-TB (58.7%), compared with DS-TB (33.1%). This is consistent with previous research demonstrating a greater prevalence of COPD among successfully treated MDR-TB patients compared to patients treated for DS-TB and community controls [159]. This supports the notion of integration of DR-TB programs with respiratory health care. Importantly, there are currently no international guidelines that recommend screening for respiratory impairment after TB treatment, although there is interest in this from several clinical and public health groups [160]. Post TB treatment respiratory care including outpatient pulmonary rehabilitation may be beneficial for some TB survivors, especially in countries with a high burden of DR-TB. DR-TB treatment completion may be a possible entry point into these programs, where they exist. Similarly, national TB programs may want to consider how they provide incentives and enablers to patients with DR-TB so that they can monitor patients closely, or consider how they can link patients to services such as social protection schemes and disability services. Both are key interventions included in the End TB Strategy [161] and in many national TB strategic plans. While incentives and enablers are frequently provided, linkages to social protection and disability services are less frequently implemented.

### Mental health disorders

Mental health disorders have historically been neglected as a focus in TB research [162–164]. Our review revealed a high prevalence of mental health disorders such as depression, anxiety and mood disorders, post-traumatic stress disorder (PTSD), and psychosis among TB patients, with substantial variation by country income level. The highest prevalence of mental health disorders was reported in LICs (42%) and the lowest prevalence in HICs (4.3%). Although our study did not include comparison data for the general population, based on other literature, we note that the prevalence of mental health disorders among TB patients in our review is higher than the prevalence of mental health disorders among the general population [165]. The prevalence of mental health disorders in our review was similar to the prevalence of mental health disorders among people with other chronic diseases such as HIV infection [166], diabetes mellitus [167], and cancer [168], from previous systematic reviews.

The relationship between mental health disorders and TB may be specific to the socioeconomic context or other factors such as health care affordability. Also, it has been well documented that TB patients and their families frequently face stigma and discrimination [169, 170]. Depression, anxiety, and other mental health disorders could be connected to this experience of stigma, loss of identity, ongoing symptoms, and the socioeconomic consequences of TB [171]. Mental health disorders can contribute to an inability to complete TB treatment and subsequently to disability [172]. The high burden of mental health disorders associated with TB suggests that additional efforts are required to improve TB care [173, 174].

### Hearing impairment

We found hearing impairment (hearing loss) among TB survivors to be common, particularly among patients with DR-TB or after taking second-line TB medications. The prevalence of hearing impairment among patients with DR-TB was 15%, which is seven times higher than the prevalence of hearing impairment among patients with DS-TB (2.3%). The disorder of hearing in patients on second-line TB medications, such as the aminoglycosides (i.e., amikacin, kanamycin, and streptomycin), is common [36, 175]. A previously published review of aminoglycoside-induced hearing impairment among TB patients also reported a high incidence of ototoxicity (7–90%) [176]. The appropriate use of TB medications should help health care providers prevent hearing loss among patients. Therefore, WHO now recommends MDR-TB treatment without the aminoglycosides [174].

We found that the prevalence of hearing impairment was higher in HICs (59%) and UMICs (27%) compared to LMICS (11%) and LICs (5%). This could be due to differences in diagnostic methods or the availability of diagnostic (auditory) equipment in HICs and UMICs to assess hearing impairment [22]. Ascertainment and/or publication bias may also be relevant here as few studies were available from LMICs and LICs. Different audiological assessment methods were used for the diagnosis of hearing impairment in our included studies, including otoscopy, pure tone audiometry, otoacoustic product emissions, and automated auditory brainstem response testing [22]. Audiometry was not always available for all patients to assess hearing impairment at baseline, during treatment, and after completion of TB treatment, to quantify the timeline of hearing loss. As a result, it was not possible to establish the main cause of hearing loss among patients with TB in this review. However the prevalence of hearing impairment in this review (14.6%) is substantially higher than the global estimates of people with disabling hearing loss in 2018 (6.1%) [177]. It is worthwhile to highlight the definition of hearing impairment as defined in this review and the local estimates may be different [174]. The hearing impairment could have a considerable effect on the quality of life, work, and social relationships [22, 178]. Therefore, hearing assessments for TB patients receiving aminoglycosides should be included as part of the management package. In addition, rehabilitation packages for those with hearing impairment should be offered routinely [22, 179].

### Neurological impairment

The patients on the second-line injectable drugs reported more than 37% neurological impairment compared to patients without an injectable TB medication (28.5%). The most common types of TB-related neurological impairments reported in our review were paraplegia, hemiplegia, cranial nerve palsies, peripheral neuropathy, hydrocephalus, and visual loss. These neurological impairments were permanent and irreversible and therefore have long-term functional, social, economic, and psychological consequences for affected patients [180, 181]. TB of the central nervous system accounts for 5–10% of all EPTB globally, with TB meningitis, intracranial TB, and spinal TB being some of the most severe forms of TB [182, 183]. TB of the spine (or Pott’s disease) affects the intervertebral discs and adjacent vertebrae, which may result in vertebral collapse, destruction, skeletal deformities, and disability [184, 185]. In addition, compression of the spinal cord and/or nerves may result in neurologic deficits [186]. To reduce the burden of neurological deficits in children from TB meningitis, improving BCG vaccination coverage in countries with low coverage of BCG is an important intervention [187].

The findings of our review suggest a pressing need to prevent or screen for TB-related disability among TB patients and survivors. Strategies to prevent or reduce TB-related disability include improving access to health care, promoting early TB diagnosis, appropriate use of TB medications, and providing training for health care workers. Adverse event monitoring, pharmacovigilance, therapeutic drug monitoring, and providing incentives and enablers for patients for treatment adherence or compliance and report adverse events should be introduced as part of the TB treatment package. After TB treatment, care including follow-up and continued monitoring for possible disability or sequelae should be initiated urgently.

### Limitations

This systematic review has several limitations. There was large heterogeneity in the prevalence of disabilities across studies which limited our ability to conduct a meta-analysis for all type of disabilities. There was also a large amount of missing data noted in our studies; for example, nearly 25% of studies had missing data for the type of TB, and HIV status was missing for 50% of the included studies. Therefore, we were unable to include these variables in the main analysis to explore the heterogeneity in these variables. In addition, in some studies, it was not possible to determine the temporal nature of the disability (i.e., whether disability occurred before, during, or after TB treatment) because the data were collected from studies that used cross-sectional study designs. For example, more than half of the papers (53%) included in the mental health impairment were cross-sectional studies. We may have some misclassification of disability for this reason. However, we attempted to explain this by conducting subgroup analysis. Moreover, we did not include studies published in languages other than English; 63 studies were excluded for this reason. Therefore, we may be missing important studies, from high TB and DR-TB burden countries such as China, the Russian Federation, and others. Lastly, as with all meta-analyses, the validity of the results is limited by the conduct and reporting of the studies from which the data were extracted and pooled.

## Conclusions

TB-related disabilities are common affecting different body parts. The burden of TB-related disability varied by the income of country, susceptibility of TB, and second-line TB drugs. The commonly reported disabilities were respiratory impairment, hearing impairment, mental health disorders, and neurological impairment. Therefore, measures to prevent and reduce TB-related disabilities should be introduced urgently as a comprehensive TB treatment package.

## Supplementary Information

**Additional file 1.** Search strategies.

**Additional file 2.** Definitions of disability in our study.

**Additional file 3.** Variables included in the data extraction tools.

**Additional file 4.** Data analysis.

**Additional file 5: Table S1.** Quality assessment tools.

**Additional file 6: Table S2.** Quality assessment article summary table.

## Data Availability

A list of included studies has been made available. The study protocol can be accessed on PROSPERO (CRD42019147488).

## Abbreviations

BCG: Bacillus Calmette–Guerin
COPD: Chronic obstructive pulmonary disease
COVID-19: Coronavirus disease
DALYs: Disability-adjusted life years
DR: Drug-resistant
DR-TB: Drug-resistant TB
DS: Drug-sensitive
EPTB: Extra-pulmonary TB
HICs: High-income countries
HIV: Human immunodeficiency virus
LICs: Low-income countries
LMICs: Low- and middle-income countries
MDR-TB: Multidrug-resistant tuberculosis
PICO: Population, Intervention, Comparator, Outcome
PRISMA: Preferred Reporting Items for Systematic Reviews and Meta-Analyses
PROSPERO: Prospective Register of Systematic Reviews
PTSD: Post-traumatic stress disorder
WPRO: World Health Organization Regional Office for the Western Pacific
TB: Tuberculosis
UMICs: Upper middle-income countries
WHO: World Health Organization
