# Supplementary material for: Tuberculosis related disability: a systematic review and meta-analysis
Source: BMC Med. 2021 Sep 9;19:203. doi: 10.1186/s12916-021-02063-9 (PMC8426113; doi:10.1186/s12916-021-02063-9)
Supplement: Supplementary file 1 — Additional file 1. Search strategies. [file 12916_2021_2063_MOESM1_ESM.docx]

**Additional file 1**: Search strategies

*PubMed*

("Tuberculosis"[Mesh] OR "Tuberculosis"[tiab] OR Tuberculoses[tiab])

AND
(“Lung function”[tiab] OR “Lung failure”[tiab] OR Pleura[tiab] OR Sequelae[tiab] OR Sequel[tiab] OR "Lung function"[tiab] OR Meninges[tiab] OR Osteoarticular[tiab] OR "Pott’s disease"[tiab] OR "Renal failure"[tiab] OR "Liver failure"[tiab] OR "Hearing impairment"[tiab] OR Ototoxicity[tiab] OR (vision[tiab] AND loss[tiab]) OR Blindness[tiab] OR "Mental health"[tiab] OR Depression[tiab] OR Anxiety[tiab] OR "Developmental delay"[tiab] OR "Development delay"[tiab])

AND

("Epidemiologic Studies"[Mesh] OR “case-control studies”[Mesh] OR “Cohort Studies”[Mesh] OR “case control”[tiab] OR Cohort[tiab] OR “Follow up”[tiab] OR Observational[tiab] OR Longitudinal[tiab] OR Prospective[tiab] OR retrospective[tiab] OR “cross sectional”[tiab] OR “Cross-Sectional Studies”[Mesh] OR Investigated[tiab] OR Analysis[tiab] OR Statistics[tiab] OR Data[tiab] OR "statistics and numerical data"[sh] OR "epidemiology"[sh])

*Embase*

('Tuberculosis'/exp/mj OR Tuberculosis:ti,ab OR Tuberculoses:ti,ab)

AND

("Lung function":ti,ab OR "Lung failure":ti,ab OR Pleura:ti,ab OR Sequelae:ti,ab OR Sequel:ti,ab OR "Lung function":ti,ab OR Meninges:ti,ab OR Osteoarticular:ti,ab OR "Potts disease":ti,ab OR "Renal failure":ti,ab OR "Liver failure":ti,ab OR "Hearing impairment":ti,ab OR Ototoxicity:ti,ab OR (vision:ti,ab AND loss:ti,ab) OR Blindness:ti,ab OR "Mental health":ti,ab OR Depression:ti,ab OR Anxiety:ti,ab OR "Developmental delay":ti,ab OR "Development delay":ti,ab)

AND

('epidemiology'/exp OR 'controlled study'/exp OR 'cohort analysis'/exp OR "case control":ti,ab OR Cohort:ti,ab OR "Follow up":ti,ab OR Observational:ti,ab OR longitudinal:ti,ab OR Prospective:ti,ab OR retrospective:ti,ab OR "cross sectional":ti,ab OR 'Cross-Sectional Studies'/exp OR Investigated:ti,ab OR Analysis:ti,ab OR Statistics:ti,ab OR Data:ti,ab)

*Web of Science (core collection)*

(Tuberculosis OR Tuberculosis OR Tuberculoses)

AND

("Lung function" OR "Lung failure" OR Pleura OR Sequelae OR Sequel OR "Lung function" OR Meninges OR Osteoarticular OR "Pott’s disease" OR "Renal failure" OR "Liver failure" OR "Hearing impairment" OR Ototoxicity OR (vision AND loss) OR Blindness OR "Mental health" OR Depression OR Anxiety OR "Developmental delay" OR "Development delay")

AND

("Epidemiologic Studies" OR "case-control studies" OR "Cohort Studies" OR "case control" OR Cohort OR "Follow up" OR Observational OR Longitudinal OR Prospective OR retrospective OR "cross sectional" OR "Cross-Sectional Studies" OR Investigated OR Analysis OR Statistics OR Data OR epidemiology)
