# Supplementary material for: Tuberculosis related disability: a systematic review and meta-analysis
Source: BMC Med. 2021 Sep 9;19:203. doi: 10.1186/s12916-021-02063-9 (PMC8426113; doi:10.1186/s12916-021-02063-9)
Supplement: Supplementary file 2 — Additional file 2. Definitions of disability in our study. [file 12916_2021_2063_MOESM2_ESM.docx]

**Additional file 2**: Definitions of disability in our study

- *Mental health disorders* included moderate and severe depression, moderate and severe anxiety, comorbid depression and anxiety, psychosis, stigma, suicidality, suicidal attempt, suicidal ideation, severe psychological distress, and post-traumatic stress disorder.
- *Neurological function impairment* included neurological deficit, hydrocephalus, peripheral neuropathy, delayed neurological and mental development, hemiplegia, seizures, palsy, paraplegia, paralysis, motor disorders, Parkinson’s Disease and stroke.
- *Respiratory impairment* included Chronic Obstructive Pulmonary Disease (COPD), chronic lung disease, restrictive respiratory impairment, Chronic Airways Obstruction (COA), destroyed lung, lung function impairment, reduced lung volume, respiratory insufficiency, bronchiectasis, emphysema, and pulmonary fibrosis.
- *Hearing impairment* included deafness (hearing loss).
- *Musculoskeletal impairment* included spinal deformity and instability, reduced limb movement, orthopaedic deformity, spondylitis, osteomyelitis, kyphosis, and chronic arthritis.
- *Visual impairment* included blindness, colour vision impairment, visual field defects, cataracts, and visual impairment.
- *Renal impairment* included renal failure and nephrotoxicity.
- Other disabilities included hepatoxicity and endocrinopathies.
