# Supplementary material for: Tuberculosis related disability: a systematic review and meta-analysis
Source: BMC Med. 2021 Sep 9;19:203. doi: 10.1186/s12916-021-02063-9 (PMC8426113; doi:10.1186/s12916-021-02063-9)
Supplement: Supplementary file 3 — Additional file 3. Variables included in the data extraction tools. [file 12916_2021_2063_MOESM3_ESM.docx]

**Additional file 3**: Variables included in the data extraction tools

We extracted the following data from each included papers: author name, year of publication, country where the study was conducted, country income level (i.e. low, lower middle, upper middle or high income, according to the World Bank Atlas method (190)), study design, sample size, mean or median (including standard deviation and range) age, percentage of male patients, bodily site affected by TB, type of TB (DS, DR or DS and DR combined, with or without an injectable agent), TB treatment regimen (DS or DR short regimen or DR long regimen), duration of TB treatment in months, duration of follow up, percentage of patients with HIV co-infection, percentage of patients with diabetes mellitus and other co-morbidities, type of disability reported, number of patients with that disability, and timing of disability diagnosis (before, during or after TB treatment).
