# Supplementary material for: Tuberculosis related disability: a systematic review and meta-analysis
Source: BMC Med. 2021 Sep 9;19:203. doi: 10.1186/s12916-021-02063-9 (PMC8426113; doi:10.1186/s12916-021-02063-9)
Supplement: Supplementary file 4 — Additional file 4. Data analysis. [file 12916_2021_2063_MOESM4_ESM.docx]

**Additional file 4**: Data analysis

Meta-analysis was undertaken to estimate the pooled proportion of each form of disability using the inverse variance heterogeneity model. Given that the proportion of disabilities were expected to be largely heterogeneous, we defined a priori potential factors that could explain the observed variance, through a meta-regression. These factors were country income level, proportion of male patients, age, type of TB and treatment provided, TB site, percentage of patients with HIV infection, and duration of TB treatment. Analyses were conducted in MetaXL version 5.3 (EpiGear Int Pty Ltd; Sunrise Beach; Australia; <http://www.epigear.com>) and Stata/MP version 14 (StataCorp, College Station, TX). Choropleth maps were generated using ArcMap version 10.7 (ESRI, Redlands, CA).
