# Supplementary material for: Tuberculosis related disability: a systematic review and meta-analysis
Source: BMC Med. 2021 Sep 9;19:203. doi: 10.1186/s12916-021-02063-9 (PMC8426113; doi:10.1186/s12916-021-02063-9)
Supplement: Supplementary file 5 — Additional file 5: Table S1. Quality assessment tools. [file 12916_2021_2063_MOESM5_ESM.docx]

**Additional file 5**: Table S1: Quality assessment tools

| **Value** | **Description** |
| --- | --- |
| **Study Population** | |
| 1 | The study population is clearly defined (i.e. adult, children, or specific group of population) * |
| 0 | The study population is not clearly defined |
| **Representativeness of the sample** | |
| 2 | Study sample is representative of the study population (all TB patients or random sampling of TB patients) ** |
| 1 | Study sample comprises a select group of the study population (non-random sampling) * |
| 0 | No description of the sampling strategy. |
| **Ascertainment of exposure** | |
| 1 | The study clearly defines the exposure (i.e. TB, MDR-TB) * |
| 0 | The study does not clearly define the exposure |
| **Sample size (max 1 star)** | |
| 1 | Justified and satisfactory (sample size and power calculation included) * |
| 0 | Not justified |
| **Non-respondents** | |
| 1 | Comparability between respondents and non-respondents’ characteristics is established, and the response rate is satisfactory. * |
| 0 | The response rate is unsatisfactory, or the comparability between respondents and non-respondents is unsatisfactory; OR no description of the response rate or the characteristics of the responders and the non-responders. |
| **Comparability: (maximum 1 point)** | |
| 1 | Where appropriate the study acknowledges and mitigates for potential bias (i.e. when comparisons are made between different study populations results are normalized for confounders) * |
| 0 | Where appropriate the study does not acknowledge or mitigate for potential bias. |
| **Outcome: (maximum 2 points)** | |
| **Assessment of the outcome (i.e. TB related disability)** | |
| 1 | Objective assessment tools or definitive diagnostic methods. |
| 0 | No definitive assessment or diagnosis tools or self-report |
| **Statistical analysis** | |
| 1 | The statistical test used is clearly described and appropriate. Where comparisons are made between population groups, the measurement of the association is presented, including confidence intervals and the probability level (p value) * |
| 0 | The statistical test is inappropriate/not described/incomplete |
