# Supplementary material for: Tuberculosis related disability: a systematic review and meta-analysis
Source: BMC Med. 2021 Sep 9;19:203. doi: 10.1186/s12916-021-02063-9 (PMC8426113; doi:10.1186/s12916-021-02063-9)
Supplement: Supplementary file 6 — Additional file 6: Table S2. Quality assessment article summary table. [file 12916_2021_2063_MOESM6_ESM.docx]

**Additional file 6: Table S2: Quality assessment article summary table**

| References | Study Population | Representativeness | Ascertainment | Sample size | Non-response | Comparability | Assessment of the outcome | Statistical analysis | Total Score |
| --- | --- | --- | --- | --- | --- | --- | --- | --- | --- |
| Aamir (2010) | 1 | 1 | 1 | 1 | 0 | 1 | 1 | 1 | 7 |
| Akaputra (2017) | 1 | 1 | 1 | 0 | 0 | 0 | 1 | 0 | 4 |
| Akkara (2013) | 1 | 1 | 1 | 0 | 1 | 0 | 1 | 1 | 6 |
| Alper (2008) | 1 | 1 | 0 | 0 | 1 | 1 | 0 | 1 | 5 |
| Appana (2016) | 1 | 1 | 1 | 0 | 0 | 1 | 1 | 0 | 5 |
| Arnold (2017) | 1 | 1 | 1 | 0 | 1 | 1 | 1 | 1 | 7 |
| Azna (2019) | 1 | 1 | 1 | 0 | 1 | 0 | 1 | 1 | 6 |
| Baig (2010) | 1 | 0 | 1 | 0 | 0 | 0 | 1 | 0 | 3 |
| Barungi (2014) | 1 | 1 | 1 | 0 | 0 | 0 | 1 | 1 | 5 |
| Batirel (2015) | 1 | 0 | 1 | 0 | 0 | 0 | 1 | 1 | 4 |
| Benzagmout (2011) | 1 | 1 | 1 | 0 | 0 | 0 | 1 | 0 | 4 |
| Bharat (2014) | 1 | 0 | 1 | 0 | 1 | 0 | 1 | 0 | 4 |
| Bhattacharyya (2011) | 1 | 0 | 1 | 0 | 0 | 0 | 1 | 0 | 3 |
| Bloss (2010) | 1 | 2 | 1 | 1 | 1 | 0 | 1 | 1 | 8 |
| Byrne (2017) | 1 | 2 | 1 | 1 | 1 | 1 | 1 | 1 | 9 |
| Castro-Silva (2018) | 1 | 1 | 1 | 0 | 0 | 0 | 1 | 1 | 5 |
| Chen (2014) | 1 | 0 | 0 |  | 0 | 0 | 1 | 1 | 3 |
| Chin (2018) | 1 | 1 | 1 | 0 | 0 | 0 | 1 | 1 | 5 |
| Christensen (2011) | 1 | 2 | 1 | 0 | 1 | 1 | 1 | 1 | 8 |
| Cohen (2019) | 1 | 1 | 1 | 0 | 0 | 0 | 1 | 1 | 5 |
| Danielle (2019) | 1 | 1 | 1 | 1 | 1 | 0 | 1 | 1 | 7 |
| Das (2014) | 1 | 1 | 1 | 0 | 0 | 0 | 1 | 1 | 5 |
| Dasa (2019) | 1 | 1 | 0 | 0 | 0 | 0 | 1 | 1 | 4 |
| Deepak (2019) | 1 | 1 | 1 | 0 | 1 | 0 | 1 | 1 | 6 |
| Deribew (2010) | 1 | 1 | 1 | 0 | 1 | 1 | 1 | 1 | 7 |
| Duko (2015) | 1 | 1 | 1 | 1 | 0 | 1 | 1 | 1 | 7 |
| Fentie (2017) | 1 | 1 | 1 | 1 | 1 | 1 | 1 | 1 | 8 |
| Fiogbe (2019) | 1 | 2 | 1 | 1 | 1 | 1 | 1 | 1 | 9 |
| Galhenage (2015) | 1 | 1 | 0 | 1 | 0 | 0 | 0 | 1 | 4 |
| Gandhi (2016) | 1 | 1 | 1 | 0 | 0 | 0 | 1 | 1 | 5 |
| García-Rodríguez (2013) | 0 | 0 | 0 | 1 | 0 | 1 | 1 | 1 | 4 |
| Garg (200) | 1 | 1 | 1 | 0 | 0 | 0 | 1 | 0 | 4 |
| Garg (2010) | 0 | 0 | 0 | 0 | 1 | 1 | 1 | 0 | 3 |
| Ghafari (2015) | 1 | 1 | 1 | 0 | 0 | 0 | 1 | 1 | 5 |
| Godoy (2012) | 1 | 1 | 1 | 0 | 0 | 0 | 1 | 1 | 5 |
| Gong (2018) | 1 | 2 | 1 | 0 | 1 | 1 | 1 | 1 | 8 |
| Gunasekeran (2018) | 1 | 1 | 1 | 0 | 1 | 0 | 1 | 1 | 6 |
| Gunawardhana (2012) | 1 | 2 | 1 | 1 | 0 | 0 | 1 | 1 | 7 |
| Gupte (2019) | 1 | 0 | 0 | 0 | 0 | 0 | 1 | 1 | 3 |
| Hadadi (2010) | 1 | 1 | 1 | 0 | 0 | 0 | 1 | 1 | 5 |
| Harouna (2019) | 1 | 2 | 1 | 1 | 0 | 0 | 1 | 1 | 7 |
| Heuvel (2013) | 1 | 1 | 1 | 1 | 0 | 0 | 1 | 1 | 6 |
| Hoa (2015) | 1 | 1 | 1 | 0 | 1 | 0 | 1 | 1 | 6 |
| Hsia (2014) | 1 | 2 | 1 | 0 | 0 | 0 | 1 | 1 | 6 |
| Hsiu-Ling (2015) | 1 | 1 | 1 | 0 | 1 | 0 | 1 | 1 | 6 |
| Hwang (2014) | 1 | 1 | 1 | 0 | 0 | 0 | 1 | 1 | 5 |
| Ige (2011) | 1 | 0 | 1 | 0 | 1 | 0 | 1 | 1 | 5 |
| Issa (2009) | 1 | 1 | 1 | 0 | 1 | 0 | 1 | 1 | 6 |
| Jianmin (2018) | 1 | 1 | 1 | 0 | 0 | 0 | 1 | 1 | 5 |
| Jo (2017) | 1 | 1 | 1 | 0 | 0 | 0 | 1 | 1 | 5 |
| Jung (2015) | 1 | 2 | 1 | 1 | 1 | 1 | 1 | 1 | 9 |
| Justin (2018) | 1 | 2 | 1 | 1 | 1 | 0 | 1 | 0 | 7 |
| Kalita (2007) | 1 | 1 | 1 | 0 | 0 | 0 | 1 | 1 | 5 |
| Kamara (2012) | 1 | 1 | 0 | 0 | 0 | 1 | 1 | 1 | 5 |
| Kamara (2012) | 1 | 1 | 1 | 1 | 1 | 0 | 1 | 1 | 7 |
| Karande (2005) | 1 | 1 | 1 | 0 | 0 | 0 | 1 | 1 | 5 |
| Kaukab (2015) | 1 | 2 | 1 | 0 | 1 | 0 | 1 | 1 | 7 |
| Kehbila (2016) | 1 | 2 | 1 | 1 | 0 | 0 | 1 | 1 | 7 |
| Khan (2018) | 1 | 1 | 1 | 0 | 1 | 0 | 1 | 1 | 6 |
| Khoza-Shangase (2016) | 1 | 1 | 1 | 0 | 1 | 0 | 1 | 1 | 6 |
| Kittikraisak (2008) | 1 | 1 | 1 | 0 | 0 | 0 | 1 | 1 | 5 |
| Lam (2010) | 1 | 1 | 1 | 1 | 0 | 0 | 1 | 1 | 6 |
| Lee (2003) | 1 | 1 | 1 | 0 | 0 | 0 | 1 | 1 | 5 |
| Lee (2017) | 1 | 1 | 1 | 1 | 1 | 1 | 1 | 1 | 8 |
| Lima (2006) | 1 | 1 | 1 | 0 | 0 | 0 | 1 | 1 | 5 |
| Lisha (2012) | 1 | 1 | 1 | 1 | 0 | 0 | 1 | 1 | 6 |
| Lucena (2015) | 1 | 0 | 1 | 0 | 1 | 0 | 1 | 1 | 5 |
| Luo (2018) | 1 | 1 | 1 | 1 | 0 | 1 | 1 | 1 | 7 |
| Luo M (2017) | 1 | 0 | 1 | 0 | 1 | 0 | 0 | 1 | 4 |
| Maguire (2009) | 1 | 1 | 1 | 0 | 0 | 0 | 1 | 1 | 5 |
| Manji (2016) | 1 | 1 | 1 | 0 | 0 | 0 | 1 | 1 | 5 |
| Masumoto (2014) | 1 | 1 | 1 | 0 | 0 | 0 | 1 | 1 | 5 |
| Maydell (2010) | 1 | 0 | 1 | 0 | 1 | 0 | 1 | 1 | 5 |
| Miftode (2015) | 1 | 1 | 0 | 0 | 0 | 1 | 1 | 1 | 5 |
| Mkoko (2019) | 1 | 1 | 1 | 0 | 0 | 0 | 1 | 1 | 5 |
| Morrone (2007) | 1 | 1 | 1 | 0 | 0 | 0 | 1 | 0 | 4 |
| Mukati (2016) | 1 | 1 | 1 | 1 | 0 | 0 | 1 | 1 | 6 |
| Nataprawira (2016) | 1 | 1 | 1 | 0 | 1 | 0 | 1 | 1 | 6 |
| Ngahane (2015) | 1 | 1 | 1 | 0 | 1 | 0 | 1 | 1 | 6 |
| Nihues (2015) | 1 | 1 | 1 | 0 | 0 | 0 | 1 | 1 | 5 |
| Njoku (2007) | 1 | 1 | 1 | 0 | 0 | 0 | 1 | 0 | 4 |
| Panda (2016) | 1 | 1 | 1 | 0 | 0 | 0 | 1 | 1 | 5 |
| Pardal (2015) | 1 | 1 | 1 | 0 | 0 | 0 | 1 | 1 | 5 |
| Park (2018) | 1 | 1 | 1 | 0 | 0 | 0 | 1 | 1 | 5 |
| Patil (2018) | 1 | 1 | 1 | 0 | 0 | 0 | 1 | 1 | 5 |
| Paulsrud (2019) | 1 | 1 | 1 | 0 | 0 | 0 | 1 | 0 | 4 |
| Peltzer (2013) | 1 | 1 | 1 | 0 | 1 | 0 | 1 | 1 | 6 |
| Piparva (2018) | 1 | 1 | 1 | 0 | 1 | 0 | 1 | 1 | 6 |
| Prakash (2017) | 1 | 1 | 1 | 0 | 0 | 0 | 1 | 1 | 5 |
| Prasad (2016) | 1 | 1 | 1 | 0 | 0 | 0 | 1 | 1 | 5 |
| Quereshi (2013) | 1 | 1 | 1 | 0 | 0 | 0 | 1 | 0 | 4 |
| Radovic (2016) | 1 | 1 | 1 | 0 | 1 | 1 | 1 | 1 | 7 |
| Ramos (2006) | 1 | 1 | 1 | 1 | 0 | 1 | 1 | 1 | 7 |
| Rastogi (2017) | 1 | 1 | 1 | 0 | 0 | 0 | 1 | 0 | 4 |
| Rhee (2014) | 1 | 1 | 0 | 0 | 0 | 0 | 1 | 1 | 4 |
| Ribeiro (2015) | 1 | 1 | 1 | 0 | 0 | 0 | 1 | 0 | 4 |
| Sagwa (2015) | 1 | 1 | 1 | 0 | 1 | 1 | 1 | 1 | 7 |
| Salodia (2019) | 1 | 1 | 1 | 1 | 0 | 0 | 1 | 1 | 6 |
| Samuel (2011) | 1 | 2 | 1 | 1 | 0 | 0 | 1 | 1 | 7 |
| Santos (2016) | 1 | 2 | 1 | 1 | 0 | 0 | 1 | 1 | 7 |
| Santra (2017) | 1 | 1 | 1 | 0 | 0 | 0 | 1 | 1 | 5 |
| Seddon (2013) | 1 | 2 | 1 | 1 | 1 | 1 | 1 | 1 | 9 |
| Sezgi (2014) | 1 | 1 | 1 | 0 | 0 | 0 | 1 | 0 | 4 |
| Shaikh (2012) | 1 | 1 | 1 | 0 | 0 | 0 | 1 | 1 | 5 |
| Sharma (2016) | 1 | 1 | 1 | 0 | 0 | 0 | 1 | 1 | 5 |
| Shean (2013) | 1 | 1 | 1 | 0 | 1 | 0 | 1 | 1 | 6 |
| Shen (2014) | 1 | 1 | 1 | 0 | 0 | 0 | 1 | 1 | 5 |
| Shen (2014) | 0 | 0 | 0 | 1 | 0 | 1 | 1 | 1 | 4 |
| Shen (2016) | 0 | 0 | 1 | 1 | 0 | 1 | 1 | 1 | 5 |
| Sheu (2010) | 0 | 1 | 0 | 0 | 0 | 1 | 1 | 1 | 4 |
| Shibeshi (2019) | 1 | 2 | 1 | 0 | 1 | 0 | 1 | 1 | 7 |
| Shyamala (2018) | 0 | 1 | 1 | 0 | 0 | 0 | 1 | 0 | 3 |
| Singla (2009) | 1 | 2 | 1 | 0 | 1 | 1 | 1 | 1 | 8 |
| Singla (2018) | 1 | 0 | 1 | 0 | 0 | 0 | 1 | 1 | 4 |
| Soriano-Arandes (2019) | 1 | 1 | 1 | 0 | 1 | 0 | 1 | 1 | 6 |
| Soumyava (2014) | 1 | 2 | 1 | 1 | 0 | 0 | 1 | 1 | 7 |
| Sun (2018) | 1 | 1 | 1 | 1 | 0 | 0 | 1 | 1 | 6 |
| Synmon (2017) | 1 | 1 | 1 | 0 | 1 | 0 | 1 | 1 | 6 |
| Tariq (2018) | 1 | 1 | 1 | 1 | 0 | 0 | 1 | 1 | 6 |
| Tinsa (2019) | 1 | 0 | 1 | 0 | 1 | 0 | 1 | 1 | 5 |
| Tomita (2019) | 1 | 1 | 1 | 0 | 1 | 0 | 1 | 1 | 6 |
| Trebucq (2018) | 1 | 1 | 1 | 0 | 0 | 0 | 1 | 1 | 5 |
| Urzua (2017) | 1 | 1 | 1 | 0 | 1 | 0 | 1 | 1 | 6 |
| Van der Walt (2013) | 1 | 1 | 1 | 0 | 1 | 1 | 1 | 1 | 7 |
| Vasconselos (2017) | 1 | 1 | 1 | 0 | 0 | 0 | 1 | 1 | 5 |
| Vashakidze (2019) | 1 | 1 | 1 | 0 | 0 | 1 | 1 | 1 | 6 |
| Wagaskar (2016) | 1 | 1 | 1 | 1 | 1 | 1 | 1 | 0 | 7 |
| Wani (2008) | 1 | 1 | 1 | 0 | 0 | 0 | 1 | 1 | 5 |
| Xavier (2015) | 1 | 1 | 1 | 0 | 0 | 0 | 1 | 1 | 5 |
| Xu (2017) | 1 | 2 | 1 | 1 | 1 | 1 | 1 | 1 | 9 |
| Yilmaz (2016) | 1 | 1 | 1 | 0 | 0 | 0 | 1 | 0 | 4 |
